# Cadence (steps/min) and relative intensity in 61 to 85-year-olds: the CADENCE-Adults study

**DOI:** 10.1186/s12966-023-01543-w

**Published:** 2023-11-29

**Authors:** Cayla R. McAvoy, Taavy A. Miller, Elroy J. Aguiar, Scott W. Ducharme, Christopher C. Moore, John M. Schuna, Tiago V. Barreira, Colleen J. Chase, Zachary R. Gould, Marcos A. Amalbert-Birriel, Stuart R. Chipkin, John Staudenmayer, Catrine Tudor-Locke, Agnes Bucko, Jose Mora-Gonzalez

**Affiliations:** 1 College of Health and Human Services, University of North Carolina at Charlotte, 9201 University City Blvd, Charlotte, NC 28223 USA; 2 Department of Kinesiology, The University of Alabama, Tuscaloosa, AL USA; 3 Department of Kinesiology, California State University, Long Beach, Long Beach, CA USA; 4 Department of Epidemiology, University of North Carolina at Chapel Hill, Chapel Hill, NC USA; 5 School of Biological and Population Health Sciences, Oregon State University, Corvallis, OR USA; 6 Exercise Science Department, Syracuse University, Syracuse, NY USA; 7 Department of Kinesiology, University of Massachusetts Amherst, Amherst, MA USA; 8 Department of Mathematics and Statistics, University of Massachusetts Amherst, Amherst, MA USA; 9 Department of Physical Education and Sports, Faculty of Sport Sciences, Sport and Health University Research Institute (iMUDS), University of Granada, Granada, Spain

**Keywords:** Accelerometer, Walking, Exercise, Physical activity, Older adults, Stepping rate

## Abstract

**Background:**

We previously demonstrated that a heuristic (i.e., evidence-based, rounded yet practical) cadence threshold of ≥ 100 steps/min was associated with absolutely-defined moderate intensity physical activity (i.e., ≥ 3 metabolic equivalents [METs]) in older adults 61–85 years of age. Although it was difficult to ascertain achievement of absolutely-defined vigorous (6 METs) intensity, ≥ 130 steps/min was identified as a defensible threshold for this population. However, little evidence exists regarding cadence thresholds and *relatively*-defined moderate intensity indicators, including ≥ 64% heart rate [HR] maximum [HR_max_ = 220-age], ≥ 40% HR reserve [HRR = HR_max_-HR_resting_], and ≥ 12 Borg Scale Rating of Perceived Exertion [RPE]; or vigorous intensity indicators including ≥ 77%HR_max_, ≥ 60%HRR, and ≥ 14 RPE.

**Purpose:**

To analyze the relationship between cadence and relatively-defined physical activity intensity and identify relatively-defined moderate and vigorous heuristic cadence thresholds for older adults 61–85 years of age.

**Methods:**

Ninety-seven ostensibly healthy adults (72.7 ± 6.9 years; 49.5% women) completed up to nine 5-min treadmill walking bouts beginning at 0.5 mph (0.8 km/h) and progressing by 0.5 mph speed increments (with 2-min rest between bouts). Directly-observed (and video-recorded) steps were hand-counted, HR was measured using a chest-strapped monitor, and in the final minute of each bout, participants self-reported RPE. Segmented mixed model regression and Receiver Operating Characteristic (ROC) curve analyses identified optimal cadence thresholds associated with relatively-defined moderate (≥ 64%HR_max_, ≥ 40%HRR, and ≥ 12 RPE) and vigorous (≥ 77%HR_max_, ≥ 60%HRR, and ≥ 14 RPE) intensities. A compromise between the two analytical methods, including Youden’s Index (a sum of sensitivity and specificity), positive and negative predictive values, and overall accuracy, yielded final heuristic cadences.

**Results:**

Across all relatively-defined moderate intensity indicators, segmented regression models and ROC curve analyses identified optimal cadence thresholds ranging from 105.9 to 112.8 steps/min and 102.0-104.3 steps/min, respectively. Comparable values for vigorous intensity indicators ranged between126.1-132.1 steps/min and 106.7–116.0 steps/min, respectively. Regardless of the relatively-defined intensity indicator, the overall best heuristic cadence threshold aligned with moderate intensity was ≥ 105 steps/min. Vigorous intensity varied between ≥ 115 (greater sensitivity) or ≥ 120 (greater specificity) steps/min.

**Conclusions:**

Heuristic cadence thresholds align with relatively-defined intensity indicators and can be useful for studying and prescribing older adults’ physiological response to, and/or perceived experience of, ambulatory physical activity.

**Trial registration:**

Clinicaltrials.gov NCT02650258. Registered 24 December 2015.

**Supplementary Information:**

The online version contains supplementary material available at 10.1186/s12966-023-01543-w.

## Introduction

The multiple benefits of a physically active lifestyle are perhaps most apparent in older adults (i.e., those > 60 years of age) who are experiencing declining physiological, cognitive, and functional trajectories associated with aging [1]. Public health guidelines typically recommend walking as an accessible, practical, and popular mode of physical activity [2]. These guidelines also recognize the salience of expressing physical activity prescriptions in terms that address volume and intensity. Traditionally, physical activity volume has been conveyed by either combining or distinguishing frequency and duration elements (e.g., 150 min/week of moderate intensity physical activity or 30 min/day, five days each week) [2]. Importantly, a step is the fundamental motor basis for ambulatory behaviors, and the popularity of walking as a mode of physical activity in older adults is increasing along with the proliferation of commercially available step-counting wearable technologies. Thus, it is not surprising that public health guidelines are recognizing the need to craft messages in terms of step counts and cadence (steps/min) [3]. As we have previously asserted, the benefits of accumulating a high daily volume of steps are generally acknowledged [3]. While research indicates that the intensity of walking (e.g., ability to walk at a self-determined brisk pace) can distinguish physical capacity in older adults [4], the use of cadence as an indicator of various physical activity intensities (i.e., relative or absolute, moderate or vigorous) is in its early stages. Guidelines supported by comprehensive analyses for older adults have been lacking and warrant attention.

The way researchers quantify the intensity of physical activity (i.e., light, moderate, or vigorous intensity) is not straightforward; for example, it can be defined in either absolute or relative terms. Absolutely-defined intensity is often communicated in metabolic equivalents standardized by body mass (METs; 1 MET = 3.5 mL·kg^− 1^·min^− 1^ of O_2_ uptake), whereas relatively-defined intensity can be expressed as a percentage of maximal oxygen uptake (%VO_2max_), percentage of heart rate maximum (%HR_max_), or percentage of heart rate reserve (%HRR). Alternatively, relative intensity can be conveyed using the Borg rating of perceived exertion (RPE) scale, which attempts to represent the subjective experience of enacted intensity [5]. Absolute definitions of physical activity are more appropriate for public health guidelines targeted for simple use by the general population, whereas relative definitions are more suitable for individualized and/or clinical exercise prescriptions [6]. In previous CADENCE-Adults reports studying adults 21–85 years of age [7–9], we presented ≥ 100 steps/min as a heuristic (rounded, evidence-based, informative, practical) cadence threshold corresponding with absolutely-defined moderate (3 METs) intensity. In this same study, a heuristic cadence threshold of ≥ 130 steps/min corresponded with absolutely-defined vigorous (6 METs) intensity in adults 21–60 years, but the evidence was weaker for 61–85 year old adults as relatively few older adults actually achieved this intensity level [9]. In a separate CADENCE-Adults report [6] assessing adults 21–60 years of age, we identified heuristic cadence thresholds associated with relatively-defined moderate (i.e., ≥ 64%HR_max_, ≥ 40%HRR, and ≥ 12 RPE) and vigorous (i.e., ≥ 77%HR_max_, ≥ 60%HRR, or ≥ 14 RPE) intensities. The relatively-defined moderate and vigorous intensity heuristic cadence thresholds were ≥ 120, 120, 115, and 105 steps/min and ≥ 135, 130, 125, and 120 steps/min, respectively for each of the age groups analyzed: 21–30, 31–40, 41–50, and 51–60 years.

Only three studies [10–12] inform relatively-defined heuristic cadence thresholds in adults ≥ 60 years of age. Serrano et al. [10], O’Brien et al. [11], and Yates et al. [12] presented 115 ± 10, 117, and 45.8 steps/min, respectively, as relatively-defined moderate intensity cadence thresholds, and O’Brien et al. [11] also reported 132 steps/min as a relatively-defined vigorous intensity heuristic cadence threshold. The seemingly discrepant conclusions likely result from differences in study approaches, including: (1) definitions of relatively-defined moderate intensity, (2) measurement of cadence, (3) administration of the walking protocol, and (4) data analysis. For example, and with regard to the first point, Serrano et al. [10] considered attainment of moderate intensity as ≥ 40% of VO_2reserve_ ([VO_2peak_ - resting VO_2_] * [0.40 + resting VO_2_]), while O’Brien et al. [11] used ≥ 40%MET_max_ as their attainment criteria, and Yates et al. [12] used METS_relative_ (calculated by dividing the oxygen cost at each treadmill speed by the participant’s measured resting value). Despite the health benefits of walking in older adults and the importance of using relative definitions for intensity, cadence thresholds associated with more practical and accessible intensity indicators (i.e., %HR_max_, %HRR, and RPE), without the need for laboratory-based equipment, remain unclear.

Herein, we expand upon our earlier work by providing cadence thresholds associated with relatively-defined intensity in a sex- and age-stratified sample of older adults between 61 and 85 years of age. Accordingly, we aimed to: (1) analyze the relationship between cadence and relatively-defined physical activity intensity in healthy older adults 61–85 years of age, and (2) identify heuristic cadence thresholds associated with commonly accepted indicators of relatively-defined moderate and vigorous physical activity intensity, namely, %HR_max_, %HRR, and RPE.

## Methods

### Study design and regulatory information

CADENCE-Adults was a cross-sectional, laboratory-based study of 21–85 year-old adults registered with Clinicaltrials.gov (NCT02650258) [6–9]. Data for the present study were collected from November 2018 to August 2019 in the Physical Activity and Health Laboratory at the University of Massachusetts Amherst. The study protocol was approved by the University of Massachusetts Amherst Institutional Review Board, and all participants provided signed informed consent. A summary of methods, procedures, and inclusion/exclusion criteria follows, and complete details can be found in a previous CADENCE-Adults report [7].

### Participants

One hundred ostensibly healthy older adults were enrolled in this portion of the CADENCE-Adults study. The recruitment strategy purposely included 10 men and 10 women for each 5-year age-band between 61 and 85 years (i.e., 61–65, 66–70, 71–75 years of age, etc.) to enhance generalizability for application of the findings. As previously reported [7], exclusion criteria were: current pregnancy, tobacco use, hospitalization for mental illness within the past 5 years, underweight (body mass index [BMI] < 18.5 kg/m^2^), class 3 obesity (BMI > 40 kg/m^2^), diagnosis of cardiovascular disease, a cerebral vascular accident, Stage 2 hypertension (systolic blood pressure ≥ 160 mmHg or diastolic blood pressure ≥ 100 mmHg), use of any medication which alters HR response, or having an implanted medical device. Additional details regarding the calculation of sample size, clinical safety protocols, and procedures for risk stratification are available in a previously published report [7].

### Treadmill testing procedures

All participants fasted for at least four hours before testing. A T31 Coded Transmitter chest strap (Polar Kempele, Finland) was used to continuously monitor HR, and resting HR was recorded after the participant sat quietly for five minutes. The original protocol [7] included up to twelve five-minute treadmill (Cybex 751T, Cybex International Inc, MA, USA) bouts at incrementally increasing speeds performed at a constant 0% grade, although none of the older adults in this sample attained the final bout’s speed. Tachometer-calibrated speed increased in 0.5 mph increments (0.8 km/h), with speeds ranging from 0.5 mph (0.8 km/h) to a maximum of 5.0 mph (8.0 km/h) and 2-minute standing rest periods separated each bout. RPE (6 to 20 Borg scale [5]) was solicited during the last minute of each bout. As reported previously [6–9], the test termination criteria included when the participant: (1) began to run, (2) reached > 75% of age-predicted HR_max_, (3) reported ≥ 14 RPE, or (4) volitionally discontinued the test. Unless they declined to continue or a safety concern arose, participants completed the bout during which they achieved any of these termination criteria.

### Measures and related data treatment

#### Participant characteristics and anthropometric variables

Self-reported descriptive characteristics included sex, age, and race/ethnicity. Standing height, leg length, and body weight were measured using standardized protocols [7]. BMI was calculated from body weight and standing height squared (kg/m^2^) [13].

#### Cadence

Steps were directly observed and hand-counted continuously throughout the protocol. Participants’ feet were video recorded for verification of step counts as needed. Cadence was calculated as the total counted steps per bout divided by the 5-min bout duration, a value expressed in steps/min units.

#### Relative intensity variables

**Heart rate (HR) variables**: HR_resting_ was based on the lowest observed HR value recorded during the seated rest period. To estimate steady-state HR, continuous HR data were averaged over minutes 2:45 − 3:45 and 3:45 − 4:45 of each 5-min treadmill bout. HR_max_ was calculated using the standard equation of 220 - age [14] and HRR was calculated using HR_max_ - HR_resting_. Both relatively-defined moderate and vigorous intensities were classified using the American College of Sports Medicine (ACSM) Guidelines for Exercise Testing and Prescription [14]. Specifically, HR-derived moderate intensity indicators were ≥ 64%HR_max_ [100 * (HR/HR_max_)] and ≥ 40%HRR [100 * (HR - HR_resting_) / (HR_max_ - HR_resting_)]. HR-derived vigorous intensity indicators were defined as ≥ 77%HR_max_ and ≥ 60%HRR.

##### Rate of Perceived Exertion (RPE)

Also as per ACSM guidelines, relatively-defined intensity was interpreted using ≥ 12 RPE (i.e., “fairly light to somewhat hard”) as moderate intensity and ≥ 14 RPE (“somewhat hard to very hard”) as vigorous intensity [14]. Previous studies confirm that older adults can reliably use the RPE scale to estimate their exertion levels during various forms of physical activity, including walking. While subjective, this scale is regularly considered a valid measure of intensity due to its correlation with physiological responses such as HR [15–17].

### Analytic sample

Data from three participants were excluded from this analysis due to a HR chest strap equipment malfunction (*n* = 1) or safety concerns identified as unsteadiness during normal ambulation (*n* = 2). Of the 97 remaining participants, test termination reasons included when the participant: (1) began to run (*n* = 3; 3.1%); (2) reached > 75%HR_max_ (*n* = 68; 70.1%); (3) reported an RPE ≥ 14 (*n* = 22; 22.7%); (4) volitionally discontinued the test, (*n* = 2; 2.1%); and/or (5) discontinued testing for safety reasons as determined by study staff (*n* = 2; 2.1%). Running and walking are distinctly different in terms of muscle activation and gait pattern [18]. Therefore, following previous CADENCE-Adults research [6–9], the very few running bouts performed (*n* = 3 in total, < 0.005% of all bouts) were excluded.

Of note, and in contrast to our experiences testing 21–60 year old adults [6], we observed that some older adults in this study chose to adopt an abnormal gait pattern during the slowest speeds (i.e., at 0.5 mph, *n* = 6 walked at ≥ 100 steps/min; at 1.0 mph, *n* = 11 walked at ≥ 100 steps/min), effectively taking very short, quick steps. As a result, some participants logged an unexpectedly high cadence early on in the testing protocol. Others seemed to adopt a strategy of taking very slow and exaggeratedly long steps for these same very slow treadmill speeds. These markedly different approaches resulted in a wide variation of cadences at the slower speeds (i.e., at 0.5 mph, cadences ranged from 38.0 to 131.4 steps/min; see Additional File 1). While these cadences increase the variability in our data, they were not considered outliers. These observations were not a result of data entry error, but rather, are legitimate data points representing natural variation in the older adult population being studied, and thus they were retained for analysis. These behavioral strategies may have been implemented to maintain balance at the slowest speed, and the older group’s relatively higher exercise HR (also Additional File 1) at these speeds were likely tied to these strategies. We did not directly measure balance during this test so our explanation for this anomaly is speculative.

### Statistical analysis

Following analytical approaches established in our previously published and related reports [6–9], we confirmed a non-linear relationship and subsequently used a segmented (bi-linear or piecewise) regression model to quantify the cadence-intensity relationship. Also aligned with preceding CADENCE-Adults reports [6–9], the breakpoint of the model was selected as that minimizing the mean squared error. The segmented regression model was fitted with both fixed effects and random intercepts by participant, and marginal R^2^ values were obtained. We assessed the potential effects of sex, age, height, leg length, and BMI by including them as control variables in separate and individual segmented regression models [11]. A forward, single stepwise regression process was implemented and the marginal R^2^ value for each analysis was interpreted to see if the covariate improved the overall prediction of the model. Again, consistent with our preceding analyses [6–9], the segmented regression equation with the 95% prediction intervals (PIs) solved for optimal cadence thresholds corresponding to each relatively-defined moderate and vigorous intensity indicator.

A ROC curve analysis was used to estimate cadence-based classifications of reaching relatively-defined moderate intensity or vigorous intensity with binary coding (i.e., meets intensity threshold/does not meet intensity threshold). Intentionally congruent with our previous reports [6–9], optimal cadence thresholds were selected as those that maximized Youden’s Index (i.e., a sum of sensitivity and specificity minus one [19, 20]). Area under the curve (AUC) values were calculated by using the bootstrap method with 20,000 replicates and 99% confidence intervals (CIs) for each of the six distilled optimal cadence thresholds [21].

Classification accuracy of each regression- and ROC-identified optimal cadence threshold for each intensity indicator (%HR_max_, %HRR, RPE) was determined as described in a previous CADENCE-Adults report [6]. Briefly, walking bouts meeting or exceeding the identified optimal cadence threshold and also meeting or exceeding the respective intensity indicator were classified as true positives (TP), while bouts that were below the identified optimal cadence threshold and also below the respective intensity indicator were classified as true negatives (TN). False positives (FP) and false negatives (FN) classifications were bouts in which the identified optimal cadence threshold was incorrect in classifying intensity. Sensitivity, specificity, positive predictive value (PPV = TP / [TP + FP]), negative predictive value (NPV = TN / [TN + FN]), and overall accuracy ([TP + TN] / [TP + TN + FP + FN]) for each regression- and ROC-identified optimal cadence threshold were assessed to ultimately inform heuristic thresholds.

#### Heuristic thresholds

The two analytical methods (i.e., regression and ROC curve) were each applied to derive the twelve cadence thresholds, one for each intensity indicator per analytic method, for a total of six thresholds for moderate intensity, and six for vigorous intensity. In keeping with our previous reports [6–9], finalized optimal heuristic cadence thresholds (i.e., rounded multiples of 5 steps/min) were determined based on an *a priori* systematic reconciliation process considering the trade-offs in terms of Youden’s Index, sensitivity, specificity, PPV, NPV, and overall accuracy. Challenging reconciliations were ultimately resolved by prioritizing those cadences that favored FN (i.e., correct classification of intensity level but at a cadence that is not the heuristic threshold), as opposed to FP classifications (i.e., incorrect classification of intensity level at the identified heuristic threshold), as well as those that maximized Youden’s Index.

## Results

### Descriptive characteristics

The analytical dataset and corresponding data dictionary are included as Additional files 2 and 3 and formatted following relevant and previously published CADENCE-Adults reports [6–9]. The dataset included 97 older adults (72.7 ± 6.9 years; 50.5% men) and 550 treadmill bouts. Descriptive statistics were expressed as means with standard deviations or counts and percentages as appropriate (Table 1). Bout characteristics (i.e., sample size, mean cadences, and relative intensity indicators) measured at each treadmill speed are summarized in Table 2.

**Table 1:** Descriptive characteristics of older adult sample (61–85 years of age)

Variable	Value
N (% male)	97 (50.5)
Age (years)	72.7 (6.9)
Height (cm)	167.2 (8.5)
Leg Length (cm)	79.9 (5.2)
Body Weight (kg)	72.5 (12.6)
BMI (kg/m^2^)	25.8 (3.5)
BMI Classification (%)	
Normal (18.5–24.9 kg/m^2^)	43 (44.3)
Overweight (25.0-29.9 kg/m^2^)	44 (45.4)
Obese (≥ 30 kg/m^2^)	10 (10.3)
Race/ethnicity (%)	
White	83 (85.6)
Black or African-American	1 (1.0)
Hispanic	0
Asian	1 (1.0)
American Indian	2 (2.1)
Unknown/No response	9 (9.3)
More than one race	1 (1.0)

Values are means and standard deviation or percentages, or counts, as appropriate. BMI = Body Mass Index (kg/m^2^) [13]

**Table 2:** Sample sizes, cadences, % heart rate maximum (HR_max_), % heart rate reserve (HRR), and RPE for treadmill speeds

		Treadmill Speed (mph)								
		**0.5**	**1**	**1.5**	**2**	**2.5**	**3**	**3.5**	**4**	**4.5**
***n***		97	87	83	79	73	65	47	17	2
**Cadence**		69.4 ± 18.9	80.7 ± 14.2	90.7 ± 11.0	98.7 ± 8.5	106.3 ± 7.1	114.2 ± 6.6	121.2 ± 7.9	129.4 ± 8.3	133.1 ± 6.1
		(38.0-131.4)	(50.6-120.2)	(66.4-125.2)	(81.4-129.8)	(93.4–128)	(100.6-131.8)	(106.2-143.6)	(112.6-148.4)	(128.8-137.4)
**%HR**_**max**_		57.2 ± 10.8	56.5 ± 8.4	57.8 ± 8.1	58.7 ± 7.6	61.2 ± 7.6	65.3 ± 7.4	69.7 ± 6.9	73.5 ± 6.9	82.5 ± 2.3
		(35.1–89.2)	(35.3–76.6)	(36.1–76.7)	(38.1–80.1)	(40.1–78.2)	(43.1–85.4)	(47.4–81)	(54.6–85.8)	(80.9–84.2)
**%HRR**		27.5 ± 14.0	27.0 ± 10.1	29.8 ± 9.9	31.6 ± 9.9	35.6 ± 9.7	43.0 ± 10.1	51.3 ± 9.9	57.9 ± 9.1	71.4 ± 3.7
		(3.9–76.5)	(5.8–59.9)	(7.9–61.2)	(13.1–66.9)	(19.0-59.6)	(25.1–72.5)	(31.4–68.5)	(40.8–77.4)	(68.8–74)
**RPE**		9.1 ± 2.2	9.5 ± 2.1	10.0 ± 2.1	10.5 ± 2.0	10.9 ± 2.0	11.8 ± 1.8	12.5 ± 1.7	13.9 ± 1.6	13.5 ± 3.5
		(6–13)	(6–16)	(6–15)	(6–15)	(6–14)	(7–15)	(7–15)	(9–15)	(11–16)

Values are means ± standard deviation with minimum and maximum in parentheses. HR maximum (HR_max_) = 220 - age. Heart rate reserve (HRR) = HR_max_ - HR_resting_. RPE = Rate of Perceived Exertion

Eighty-eight participants reached moderate intensity defined by ≥ 64%HR_max_, 89 participants reached moderate intensity defined by ≥ 40%HRR, and 73 participants reached moderate intensity defined by ≥ 12 RPE. Sixty-four participants (71.7 ± 6.4 years of age; 48.4% women) reached all three moderate intensity indicators combined. Twenty-five participants reached vigorous intensity defined by ≥ 77%HR_max_, 24 participants reached vigorous intensity defined by ≥ 60%HRR, and 38 participants reached vigorous intensity defined by ≥ 14 RPE. Overall, 71 participants met at least one of these three indicators of vigorous intensity, and 8 participants (68.0 ± 4.5 years of age; 62.5% women) reached all three vigorous intensity indicators. Two participants (63.5 ± 2.1 years of age; 0% women) completed all 9 bouts and attained the maximum speed of 4.5 mph.

### Segmented regression model

**Fig. 1:**
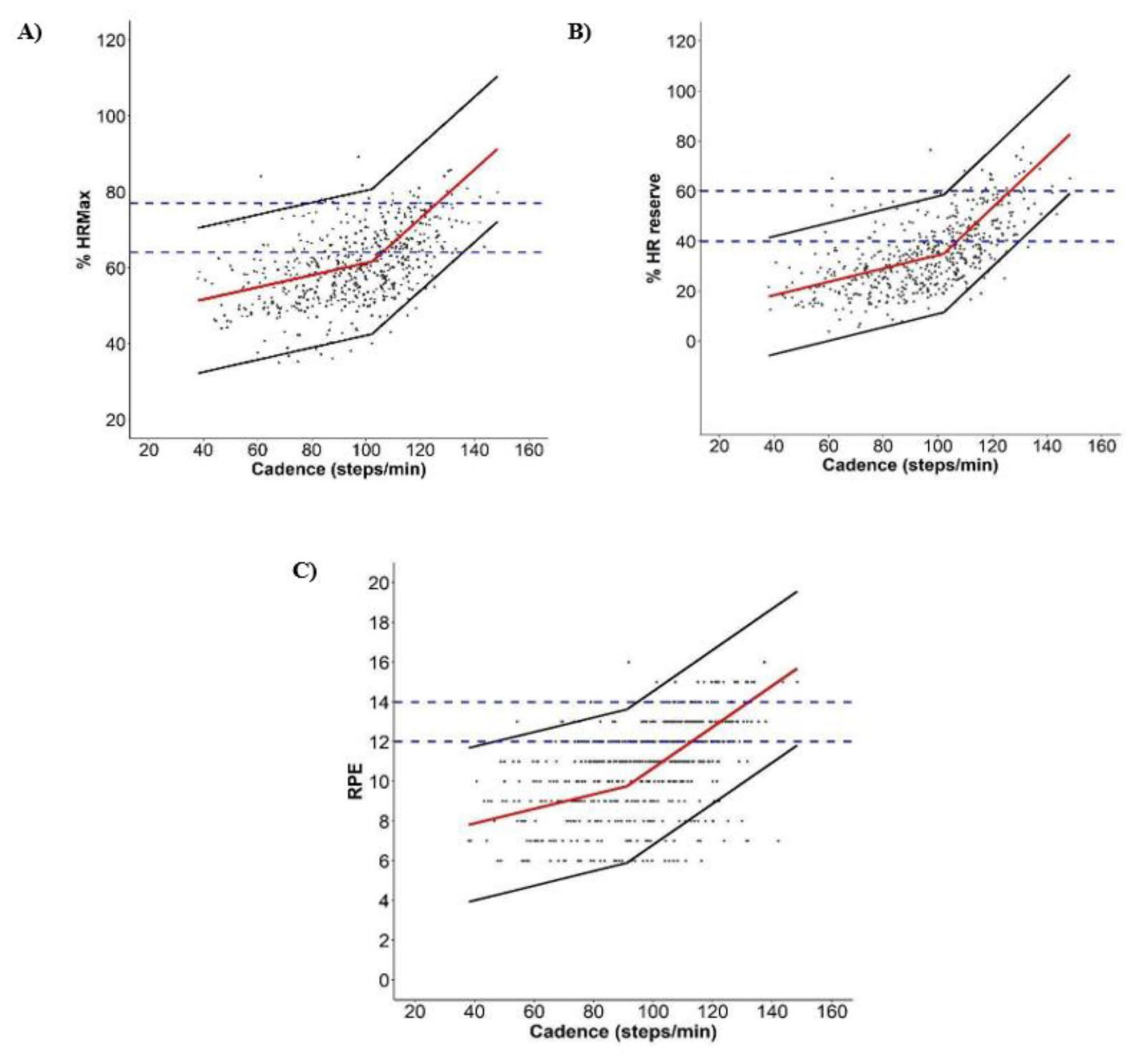
Relationship between cadence and relative intensity indicators (A) %Heart rate maximum (%HR_max_); B) %Heart rate reserve (%HRR); and C) Rate of Perceived Exertion (RPE), using a segmented regression model. Red line is the mean relative intensity value at each corresponding cadence value, and black lines are the 95% prediction intervals. Blue dotted and dash-dotted lines represent, respectively, moderate and vigorous intensity thresholds

Figure 1 shows the segmented regression models depicting the relationship between cadence and each of the relatively-defined intensity indicators. The regression breakpoints were 102.20 steps/min for %HR_max_ and %HRR, and 91.20 steps/min for RPE. The pre-breakpoint slope for %HR_max_ was 0.16 (95% CI: 0.14–0.18) and post-breakpoint slope was 0.64 (95% CI: 0.61–0.68). Accordingly, the equation to predict %HR_max_ from cadences or for intensities below the breakpoint (i.e., ≤ 102.20 steps/min or ≤ 62%HR_max_) was: $\%HRmax=45.28+0.16\times cadence$, and the equation for cadences or intensities above the breakpoint (i.e., > 102.20 steps/min or > 62%HR_max_) was: $\%HRmax \hspace{0.17em}=\hspace{0.17em}\hspace{0.17em}-\hspace{0.17em}4.05 + 0.64 \times cadence$. For %HRR, the pre-breakpoint slope was 0.27 (95% CI: 0.23–0.30) and post-breakpoint slope was 1.03 (95% CI: 0.97–1.10). The equation to predict %HRR from cadences or for intensities below the breakpoint (i.e., ≤ 102.20 steps/min or ≤ 35%HRR) was: $\%HRR =\hspace{0.17em}7.67 + 0.27 \times cadence$; and above the breakpoint (i.e., > 102.20 steps/min or > 35%HRR) was:$\%HRR \hspace{0.17em}=\hspace{0.17em}-70.71 + 1.03 \times cadence$. For RPE, the pre-breakpoint slope was 0.04 (95% CI: 0.03–0.04) and post-breakpoint slope was 0.10 (95% CI: 0.10–0.11). To predict RPE from cadences or for intensities below the breakpoint (i.e., ≤ 91.20 steps/min or ≤ 10 RPE), the equation was: $RPE\hspace{0.17em}=\hspace{0.17em}6.42 + 0.04 \times cadence$, and the equation for cadences or intensities above the breakpoint (i.e., > 91.20 steps/min or > 10 RPE) was: $RPE\hspace{0.17em}=\hspace{0.17em}0.30 + 0.10 \times cadence$. There was no substantial improvement in the magnitude of the cadence-intensity associations when sex, age, height, leg length, or BMI were included in individual models as covariates (marginal R^2^ only varied by ≤ ~ 0.05). Cadence showed the strongest association with %HRR (R^2^ = 0.45), followed by RPE (R^2^ = 0.34) and %HR_max_ (R^2^ = 0.33).

Relatively-defined moderate and vigorous intensity optimal cadence thresholds identified by the segmented regression models are described in Table 3. Across all intensity indicators, the optimal cadence thresholds (and 95% PIs) associated with moderate intensity using this specific analytical approach and defined by ≥ 64%HR_max_, ≥ 40%HRR, and ≥ 12 RPE were 105.9 (38.0-135.7), 107.1 (38.0-129.8), and 112.8 (46.6-148.4) steps/min, respectively. Corresponding values for vigorous intensity defined by ≥ 77%HR_max_, ≥ 60%HRR, and ≥ 14 RPE were 126.1 (79.0-148.4), 126.4 (103.6-148.4), and 132.1 (94.9-148.4) steps/min, respectively. Across all intensity indicators, sensitivity values were 40.1–66.3% for moderate intensity and 15.8–46.2% for vigorous intensity, whereas corresponding specificity values ranged from 78.0 to 88.3% for moderate intensity and 96.0-99.2% for vigorous intensity. PPV values were 60.2–67.5% for moderate intensity and 30.0–60.0% for vigorous intensity indicators, while NPV values were 71.0-84.8% and 94.1–97.3% for moderate and vigorous intensity indicators, respectively. Across all intensity indicators, overall accuracy was 70.2–78.2% for moderate intensity and 93.3–94.5% for vigorous intensity.

**Table 3:** Cadence thresholds (steps/min) for relatively-defined intensity indicators of moderate and vigorous intensity based on regression and ROC curve analyses

		Regression	ROC
***Moderate Intensity***			
**≥ 64% HR**_**max**_	Threshold (steps/min)	105.9 (38.0-135.7)	104.3 (97.1-110.7)
	Se	60.5	65.6
	Sp	78.0	75.8
	PPV	60.2	59.8
	NPV	78.2	80.1
	Accuracy	71.8	72.2
	AUC	–	76.7 (72.5–80.9)
**≥ 40% HRR**	Threshold (steps/min)	107.1 (38.0-129.8)	103.3 (100.8-110.7)
	Se	66.3	77.5
	Sp	83.5	75.6
	PPV	64.0	58.5
	NPV	84.8	88.3
	Accuracy	78.2	76.2
	AUC	–	83.1 (79.3–86.9)
**≥ 12 RPE**	Threshold (steps/min)	112.8 (46.6-148.4)	102.0 (95.8-104.1)
	Se	40.1	71.0
	Sp	88.3	74.9
	PPV	67.5	63.1
	NPV	71.0	81.1
	Accuracy	70.2	73.5
	AUC	–	78.2 (74.3–82.0)
***Vigorous Intensity***			
**≥ 77% HR**_max_	Threshold (steps/min)	126.1 (79.0-148.4)	116.0 (97.1-128.6)
	Se	36.0	60.0
	Sp	96.0	85.9
	PPV	30.0	16.9
	NPV	96.9	97.8
	Accuracy	93.3	84.7
	AUC	–	76.1 (65.4–86.8)
**≥ 60% HRR**	Threshold (steps/min)	126.4 (103.6-148.4)	109.7 (109.7-119.1)
	Se	46.2	92.3
	Sp	96.9	76.0
	PPV	42.9	16.0
	NPV	97.3	99.5
	Accuracy	94.5	76.7
	AUC	–	88.4 (80.7–96.1)
**≥ 14 RPE**	Threshold (steps/min)	132.1 (94.9-148.4)	106.7 (98.1-116.8)
	Se	15.8	84.2
	Sp	99.2	70.3
	PPV	60.0	17.4
	NPV	94.1	98.4
	Accuracy	93.5	71.3
	AUC	–	84.4 (78.4–90.3)

The thresholds are represented as means (95% Prediction Intervals) for segmented regression and means (99% Confidence Intervals) for Receiver Operating Characteristic (ROC) curve. Classification accuracy analyses to calculate Sensitivity (Se), Specificity (Sp), Positive Predictive Value (PPV), Negative Predictive Value (NPV) and Accuracy were performed independently on these two optimal thresholds derived from segmented regression and ROC curve analysis, therefore yielding two values for each classification accuracy metric. AUC = area under the curve. HR maximum [HR_max_] = 220 - age. Heart rate reserve [HRR] = HR_max_ - HR_resting_. RPE = Rate of Perceived Exertion

### Receiver operating characteristic analyses

ROC curve analysis results for optimal cadence thresholds related to relatively-defined intensity indicators are also displayed in Table 3. Across all intensity indicators, the optimal cadence thresholds (and 99% CIs) associated with moderate intensity using this specific analytical approach and defined as ≥ 64%HR_max_, ≥ 40%HRR, and ≥ 12 RPE were 104.3 (97.1-110.7), 103.3 (100.8-110.7), and 102.0 (95.8-104.1) steps/min, respectively. Corresponding values for vigorous intensity defined as ≥ 77% HR_max_, ≥ 60% HRR, and ≥ 14 RPE were 116.0 (97.1-128.6), 109.7 (109.7-119.1), and 106.7 (98.1-116.8) steps/min, respectively. As presented in Table 3, sensitivity ranged from 65.6 to 77.5% across moderate intensity indicators and 60.0-92.3% for vigorous intensity indicators. Specificity values ranged from 74.9 to 75.8% for moderate intensity and 70.3–85.9% for vigorous intensity. PPV values were 58.5–63.1% and 16.9–17.4% for moderate intensity and vigorous intensity indicators, respectively, while NPV values were 80.1–88.3% for moderate intensity and 97.8–99.5% for vigorous intensity indicators. Across all intensity indicators, overall accuracy was 72.2–76.2% for moderate intensity and 71.3–84.7% for vigorous intensity. AUC values were 76.7–83.1% and 76.1–88.4% for moderate and vigorous intensities, respectively.

### Heuristic thresholds

The final identified heuristic cadence thresholds for relatively-defined moderate and vigorous intensity, and respective classification accuracy metrics for correctly (TP, TN) or incorrectly (FP, FN) classifying walking bouts, are summarized in Table 4 and graphically presented in Additional file 4. The potential heuristic thresholds ultimately selected were rounded to the nearest 5 steps/min for ease of communication and application. When potential heuristic thresholds straddled two numbers divisible by five (i.e., ≥ 100 versus 105 steps/min for relatively-defined moderate intensity and ≥ 115 versus 120 steps/min for vigorous intensity), the analytical tradeoffs mentioned previously were considered. The heuristic cadence threshold selected for relatively-defined moderate intensity was ≥ 105 steps/min. The Youden’s Index associated with both ≥ 105 steps/min and ≥ 100 steps/min was the same (0.45) but the FN rate (33% versus 26%, respectively) was deemed more tolerable than the associated FP rate (23% versus 30%, respectively) when using ≥ 105 steps/min.

**Table 4:** Heuristic cadence thresholds (steps/min) for relatively-defined moderate and vigorous intensity based on segmented regression and ROC curve analyses

Intensity Level	Intensity Indicator		Measure	
**Moderate Intensity Heuristic Threshold**: **≥ 105 steps/min**	**≥ 64%HR**_**max**_		Se	63.6
			Sp	75.8
			PPV	59.0
			NPV	79.1
			Accuracy	71.5
	**≥ 40%HRR**		Se	74.0
			Sp	77.7
			PPV	59.5
			NPV	87.1
			Accuracy	76.5
	**≥ 12 RPE**		Se	64.7
			Sp	77.8
			PPV	63.8
			NPV	78.5
			Accuracy	72.9
**Vigorous Intensity Heuristic Threshold**: **≥ 115 steps/min**	**≥ 77%HR**_**max**_		Se	60.0
			Sp	82.7
			PPV	14.2
			NPV	97.7
			Accuracy	81.6
	**≥ 60%HRR**		Se	80.8
			Sp	83.8
			PPV	19.8
			NPV	98.9
			Accuracy	83.6
	**≥ 14 RPE**		Se	63.2
			Sp	84.0
			PPV	22.6
			NPV	96.8
			Accuracy	82.5
**Vigorous Intensity Heuristic Threshold**: **≥ 120 steps/min**	**≥ 77%HR**_**max**_		Se	28.2
			Sp	97.2
			PPV	84.6
			NPV	71.1
			Accuracy	72.7
	**≥ 60%HRR**		Se	61.5
			Sp	90.6
			PPV	24.6
			NPV	97.9
			Accuracy	89.3
	**≥ 14 RPE**		Se	50.0
			Sp	91.0
			PPV	29.2
			NPV	96.1
			Accuracy	88.2

Trade-offs in terms of Sensitivity (Se), Specificity (Sp), Positive Predictive Value (PPV), Negative Predictive Value (NPV), and overall accuracy between the thresholds derived from the segmented regression and the Receiver Operating Characteristic (ROC) curve analyses were considered.

Two heuristic cadence thresholds were ultimately selected for relatively-defined vigorous intensity, ≥ 115 or ≥ 120 steps/min, depending on future users’ priority needs in terms of sensitivity or specificity, respectively. Specifically, for ≥ 115 steps/min, Youden’s Index = 0.52, FN classifications = 30%, FP classifications = 20%, and 82.6% of bouts were correctly classified (accuracy, i.e., TP + TN / N). For ≥ 120 steps/min, Youden’s Index = 0.41, FN classifications = 50%, FP classifications = 10%; and 89.3% of bouts were correctly classified. Thus, despite the lower comparative Youden’s Index, ≥ 120 steps/min fit the *a priori* stated higher tolerance towards FN classifications over FP classifications and correctly classified more bouts. However, overall accuracy is a metric best suited for symmetric datasets (similar counts TP, TN, FP, and FN classifications) [18], and in this case, a higher overall accuracy was achieved by setting a higher cadence threshold. For example, a cadence threshold of ≥ 150 steps/min would have high values of TP and TN, mathematically driving a higher overall accuracy when using the equation (TP + TN)/(TP + FP + FN + TN). The dataset from our sample became increasingly imbalanced the higher the threshold (e.g. when using %HRR, FN:TN = 44:296 ≥ 115 steps/min and 10:475 for ≥ 120 steps/min). Thus, we ultimately conceded that, for older adult samples, either ≥ 115 or ≥ 120 steps/min could satisfy potentially different analytical needs in terms of sensitivity vs. specificity, respectively.

To be clear, averaged over the three vigorous-intensity indicators (≥ 77%HR_max_, ≥ 60%HRR, and ≥ 14 RPE), the sensitivity was 68.0 and specificity was 83.5 for ≥ 115 steps/min compared to the sensitivity of 50.5 and specificity of 90.4 for ≥ 120 steps/min. Put together, ≥ 115 steps/min fit our *a priori* prioritization of maximizing Youden’s Index and was the more sensitive heuristic threshold, while ≥ 120 steps/min fit our *a priori* stated tolerance towards favoring FN classifications over FP classifications, and was the more specific heuristic threshold.

## Discussion

A heuristic cadence threshold of ≥ 105 steps/min was ultimately identified as a useful indicator of relatively-defined moderate intensity walking (defined as ≥ 64%HR_max_, ≥ 40%HRR, or ≥ 12 RPE) in a sample of older adults 61–85 years of age. Furthermore, while we report the single heuristic threshold for moderate intensity for simplicity (≥ 105 steps/min), based on our findings, 105–114 steps/min would indicate the range of moderate intensity walking in this population, and anything above that range would indicate vigorous intensity walking.

Heuristic cadence thresholds of ≥ 115 steps/min (greater sensitivity) or ≥ 120 steps/min (greater specificity) were identified as indicators of relatively-defined vigorous intensity walking (defined as ≥ 77%HR_max_, ≥ 60%HRR, or ≥ 14 RPE). These two vigorous intensity thresholds (≥ 115 steps/min or ≥ 120 steps/min) can be useful in different practical or research scenarios. For example, ≥ 115 steps/min is more sensitive and therefore more inclusive, which would result in more older adults being classified as walking at vigorous intensity. This inclusive approach would be preferential for interventions and public health messaging. On the other hand, ≥ 120 steps/min is a more specific threshold, and therefore more exclusive. Thus, it may be preferred by researchers whose questions may require a more stringent approach to identifying older adults who are walking at vigorous intensity. Finally, highly motivated individuals subscribing to the quantified-self movement could self-select their preferred training cadence by interpreting their own physiological response and perceived experience at each of the proposed thresholds.

Previous research on this topic has reported an R^2^ of 0.34 when using %VO_2reserve_ as the intensity indicator [10] and 0.77 when using %MET_max_ as the intensity indicator [11]. The study conducted by Serrano et al. [10] examined cadence during overground walking (measured via a Garmin FR60 foot device) and relatively-defined intensity in 121 older adults (mean age = 68.6 years, 59.5% women) while defining moderate intensity as ≥ 40% of VO_2reserve_ ([VO_2peak_ - resting VO_2_] * [0.40 + resting VO_2_]) obtained using a peak aerobic fitness test [10]. That study used a linear regression algorithm and cross-validation analysis to conclude that 115 ± 10 steps/min was the best overall point index value of moderate intensity, i.e., the same value that we settled on as a heuristic threshold associated with vigorous intensity in older adults. However, the lower end of the researchers’ recommended range for moderate intensity of ≥ 105 to 125 steps/min is still congruent with our finding of a heuristic threshold value of ≥ 105 steps/min for moderate intensity.

O’Brien et al. [11] also studied the association between cadence and moderate intensity defined as ≥ 40%MET_max_ and vigorous intensity defined as ≥ 60%MET_max_ in 19 older adults (mean age = 69 years, 36.8% women). They used a progressive walking treadmill protocol with speeds spanning between 1.5 mph (2.4 km/h) and 4.0 mph (6.4 km/h). These researchers also used mixed-effects modeling to develop an equation that predicted cadence at moderate and vigorous intensity and ROC curve analysis to evaluate potential cadence thresholds maximizing Youden’s Index. They concluded that 117 steps/min and 132 steps/min aligned with relatively-defined moderate and vigorous intensity, respectively, and that BMI influenced the cadence-intensity relationship. In our sample, 48.5% (*n* = 47) were able to reach O’Brien et al.’s [11] threshold of ≥ 117 steps/min, but only 8.2% (*n* = 8) reached their threshold of ≥ 132 steps/min. For comparison, 79.4% (*n* = 77) walked at our threshold of ≥ 105 steps/min, 57.7% (*n* = 56) walked at ≥ 115 steps/min, and 41.2% (*n* = 40) walked at ≥ 120 steps/min. The higher cadence thresholds that O’Brien et al. [11] found might be tied to their use of different definitions of moderate and vigorous intensity compared to those applied in the current study. Further, the sample who participated in O’Brien et al.’s [11] study averaged a VO_2max_ of 31.0 ml/kg/min (standard deviation = 2.9; range = 25.7–35.7), averaging “fair” to “good” in aerobic fitness based on their completion of the Ebbeling walking treadmill protocol [22]. While testing for %VO_2max,_ %VO_2peak,_ or %MET_max_ might more accurately account for individual fitness levels, such assessments are not routinely available. Conversely, our reported thresholds are both accessible and feasible as they are based simply upon resting HR, age-predicted HR_max_, or RPE.

Another article studying cadence and intensity in older adults was recently published by Yates et al. [12], who analyzed data collected from 53 older adults (median age = 75 years, 45.3% women). These researchers derived cadence from an activPAL3 device (notably, not using direct observation as a criterion standard) and defined moderate intensity in two different ways, one of which differed from our own definitions. They calculated METS_standard_ in the same way we previously computed absolutely defined MET values in our study of absolute intensity [9] in terms of oxygen cost expressed in mL/kg/min and divided by the resting standard of 3.5 mL/kg/min. More applicable to this present analysis focused on relative intensity, their definition, METS_relative_, was calculated by dividing the oxygen cost of each treadmill speed by the participant’s measured resting value. Participants in the Yates et al. [12] study completed a randomly ordered treadmill protocol comprised of 5-minute bouts at speeds of 1, 2, 3, 4, and 5 km/h (0.6, 1.2, 1.9, 2.5, and 3.1 mi/h). Segmented generalized estimating equations were used to analyze the relationship between device-derived cadence and MET values, specifically to predict cadence at 3 METS_standard_ and 3 METS_relative_. ROC curve analysis was used to analyze the relationship between cadence and the METS_standard_ moderate intensity classifications. The Yates et al. [12] study concluded that a cadence as low as 70 steps/min corresponded to moderate intensity physical activity as defined by METS_standard_, and that the predicted cadence value at 3 METS_relative_ was 45.8 steps/min. Although Yates et al. [12] reported estimates that appear much lower than those identified in the present study, it is important to note that their 70 steps/min threshold actually falls within the prediction interval reported by the original CADENCE-Adults study (70.0–114.2 steps/min) [9], which was based on the same absolute definition of METs. The heuristic threshold of 100 steps/min in the CADENCE-Adults study was derived from subsequent analyses intended to maximize classification accuracy.

The more notable discrepancy in threshold values based on METS_relative_ is likely due to differences in definitions for moderate intensity physical activity, with Yates et al. [12] deviating from traditional definitions based on %HR_max_, HRR, or RPE. We do not know if the participants in the Yates et al. [12] study who reached 3 METS_relative_, also achieved the criteria we employed, specifically ≥ 64%HR_max_, ≥ 40%HRR, or ≥ 12 RPE. Additional methodological differences between studies may have also contributed to the discrepant results. As mentioned above, Yates et al. [12] derived an estimate of steps taken from a wearable technology, not from the criterion standard of direct observation that we employed. Wearable technologies are well known to introduce error and reduce accuracy, particularly at low speeds. For example, in the CADENCE-Adults study the activPAL demonstrated an absolute percent error of 5.67% at 1.0 mi/h (1.6 km/h) [23].

As mentioned above, a previous report from CADENCE-Adults study [6] examined cadence and relatively-defined intensity using the same intensity indicators and analyses as the current report in a sample of 157 adults (mean age = 40.4 years, 49.4% women) stratified by age groups (21–30, 31–40, 41–50, and 51–60 years). Conclusions revealed that heuristic cadence thresholds for the chronologically-arranged age groups were 120, 120, 115, and 110 steps/min across all relatively-defined moderate intensity indicators and 135, 130, 125, and 120 steps/min across all relatively-defined vigorous intensity indicators. The findings herein extend these values for 61–85 year old adults (≥ 105 for moderate and ≥ 115 or ≥ 120 steps/min, for vigorous intensity, respectively) and align expectedly with age-associated physiological and functional changes [1]. For comparative purposes and an overview across the adult lifespan, Additional file 5 concatenates our previous work to present a table with relatively-defined heuristic cadence thresholds by age range for adults 21–85 years.

As stated earlier, our previous research supports that ≥ 100 steps/min is associated with absolutely-defined moderate intensity (defined as 3 METs) in older adults [9], but herein we establish that the heuristic cadence threshold required to reach ≥ 64%HR_max_, ≥ 40%HRR, or ≥ 12 RPE, all commonly accepted indicators of *relatively*-defined moderate intensity, is slightly higher (i.e., ≥ 105 steps/min), more precisely reflecting one’s relative physiological response to, or perceived experience of, physical activity. While these values (≥ 100 and ≥ 105 steps/min) are slightly different, absolute and relative intensity are not directly comparable and have separate applications in practice and research. For instance, while the threshold of ≥ 100 steps/min can be used for evaluating absolutely-defined moderate intensity at the population level, if an adult’s age is known, then it is possible to prescribe a cadence that is more aligned with expectations for relatively-defined moderate intensity in their age group, and thus more individualized [6]. As a simple example, a patient who is 70 years of age and engaged in a cardiac rehabilitation program could be prescribed a training cadence of ≥ 100 steps/min (a conservative starting point congruent with absolutely-defined moderate intensity [9]), and progress to ≥ 105 steps/min (likely more aligned with their physiological response and/or perceived experience relative to functional capacity). Then, applying the principle of overload [14], additional incremental increases in cadence, and consequently, training intensity could be prescribed. Further, if the desire is to keep the patient below vigorous intensity (relatively-defined), a ceiling value of 114 steps/min (i.e., less than the vigorous intensity heuristic threshold of ≥ 115 steps/min) could be implemented.

We acknowledge that there are conflicting findings across relevant literature regarding the potential effects of covariates (e.g., sex, age, height, leg length, and BMI) on the cadence-intensity relationship. For example, as it relates to older adults, Serrano et al. [10] reported that height, body weight, BMI, VO_2peak_, and self-selected cadence were significantly associated with moderate intensity defined ass ≥40% of VO_2reserve_, while leg length and stride length were not. O’Brien et al. [11] reported an influence of BMI, but not height or leg length, in their sample of older adults. In contrast, we observed no substantial improvement in the magnitude of cadence-intensity associations when age was added as a covariate, despite our extensive assessment of this potential effect. Specifically, although we observed statistically significant differences in both exercise HR and cadence when stratified by age at specific lower speeds (0.5, 1.0, and 1.5 mph; p < 0.05; Additional File 1) at higher (and more natural) walking speeds (≥ 2.0mph), neither exercise HR nor cadence were significantly different (HR: range of p = 0.15–0.85, Cadence: range of p = 0.08–0.89), indicating similar physical capabilities between age groups. Despite these inconsistent findings, we incorporated age (continuously and in quartiles) as a covariate in our regression models, and considered stratification by decade of age. These adjustments did not result in substantial changes in the models’ R^2^ values or AIC/BIC values. To be specific, marginal R^2^ values changed by 0 for RPE, 0.06 for HRR, and 0.09 for HRmax, however, we considered that age is a component of the equations for HRR and HRmax. RPE, the only variable without age in the formula, saw no change. Additionally, we observed no substantial improvement in the magnitude of cadence-intensity associations when sex, height, leg length, or BMI were included in models as covariates. These discrepant findings are likely influenced by sample size and characteristics (i.e., diversity in values with regards to measured or unmeasured parameters) and/or methodology and study design (e.g., overground walking versus treadmill walking, different statistical analysis performed).”

A strength of our study is that the older adult sample encompassed a sex- and age-balanced distribution to inform heuristic cadence thresholds using three indicators of relatively-defined intensity. Additionally, we used the criterion indicator to measure cadence (direct observation) as opposed to relying on estimates of wearable technologies. However, this research should be interpreted within the context of its limitations. First, while heuristic thresholds are purposely rounded for practicality, communication, and application, this method limits precision. We believe that by applying an innovative, more individualized, age-granular surface-knitted modeling approach (e.g., locally estimated scatterplot smoothing or LOESS [24]) while using a larger sample size, future researchers will be able to address inter-age physiological differences [25] with enhanced precision. Additionally, while we tried to control for potential inter-individual differences in biological and anthropometrical factors, there is always the possibility of unassessed potential confounding variables (e.g., fitness level, frailty). While the scope of this research was to investigate the cadence-intensity relationship in independently ambulatory and ostensibly healthy older adults, it would be interesting to extend this exploration to older adults with gait impairments or those who use gait aids to ambulate who might have higher metabolic cost during walking (i.e., high energy expenditure) relative to apparently healthy older adults who might have higher metabolic cost during walking (i.e., high energy expenditure) relative to apparently healthy older adults [26]. Finally, while this original treadmill-based study was planned to provide a necessary foundation of evidence, further investigations should address the cadence-intensity relationship in non-laboratory conditions, including during overground walking and/or in free-living settings.”

## Conclusion

This study analyzed the relationship between cadence and relatively-defined physical activity intensity in healthy older adults 61–85 years of age and identified heuristic cadence thresholds associated with the intensity indicators of %HR_max_, %HRR, and RPE. A heuristic cadence threshold of ≥ 105 steps/min was associated with relatively-defined moderate intensity in older adults. With regards to relatively-defined vigorous intensity, ≥ 115 steps/min was identified as a more sensitive threshold suitable for intervention/messaging purposes and ≥ 120 steps/min was a more specific threshold that may be preferred for more stringent research questions in this age group. Overall, these findings extend our earlier research focused on relatively-defined intensity and cadence in adults 21–60 years of age [6], as well as absolutely-defined intensity and cadence in adults 21–85 years of age [7–9] to more fully capture the relationship between cadence and physical activity intensity in the many ways it is defined across the adult lifespan.

### Electronic supplementary material

Below is the link to the electronic supplementary material.

**Additional file 1.** Table displaying exercise heart rate and cadences by treadmill speed and age group

**Additional file 2**. Spreadsheet displaying final analytical dataset

**Additional file 3**. Spreadsheet displaying the data dictionary

**Additional file 4**. Figure displaying classification accuracy of heuristic cadence thresholds and relatively-defined moderate and vigorous intensity

**Additional file 5**. Table with heuristic thresholds by age ranges

## Data Availability

All data generated or analyzed during this study are included in this article and its additional files.

## Abbreviations

ACSM: American College of Sports Medicine
AUC: Area under the curve
BMI: Body mass index
CI: Confidence interval
HR: Heart rate
HRR: Heart rate reserve
HR_max_: Heart rate maximum
kg: kilogram
m^2^: meters squared
METs: Metabolic equivalents
mph: Miles per hour
NPV: Negative predictive value
PI: Prediction interval
PPV: Positive predictive value
ROC: Receiver operating characteristic
RPE: Rating of perceived exertion
TN: True negative
TP: True positive
VO_2_: Oxygen consumption
