# Supplementary material for: Cadence (steps/min) and relative intensity in 61 to 85-year-olds: the CADENCE-Adults study
Source: Int J Behav Nutr Phys Act. 2023 Nov 29;20:141. doi: 10.1186/s12966-023-01543-w (PMC10688086; doi:10.1186/s12966-023-01543-w)
Supplement: Supplementary file 1 — Additional file 1. Table displaying exercise heart rate and cadences by treadmill speed and age group [file 12966_2023_1543_MOESM1_ESM.docx]

| **Additional File 1.** Exercise HR and cadences by treadmill speed and age group. | | | | | |
| --- | --- | --- | --- | --- | --- |
| **Speed (mph)** | **Age Group (years)** | **Mean Exercise HR (bpm)** | ***P*** | **Mean Cadence (steps/min)** | ***P*** |
| All | 61-70 | 88.8 (54 - 133) | 0.10 | 94.7 (38 – 144) | 0.55 |
|  | 71-85 | 90.7 (67.3 - 125) |  | 95.8 (38.6 – 148) |  |
| **0.5** | **61-70** | **79.8 (54 - 117.9)** | **0.03** | **63 (38 - 101.4)** | **<0.01** |
|  | **71-85** | **86.6 (67.3 - 123.6)** |  | **73.7 (38.6 - 131.4)** |  |
| **1** | **61-70** | **79.7 (54.4 - 105.7)** | **0.01** | **74.9 (58.8 - 103)** | **<0.01** |
|  | **71-85** | **85.6 (71.3 - 109)** |  | **85 (50.6 - 120.2)** |  |
| **1.5** | **61-70** | **82.4 (55.6 - 107.7)** | **0.04** | **87.5 (73.2 - 106.4)** | **0.01** |
|  | **71-85** | **87.4 (73.6 - 111.2)** |  | **93.2 (66.4 - 125.2)** |  |
| 2 | 61-70 | 85.2(58.8 - 108.6) | 0.15 | **97 (84 - 112.4)** | 0.08 |
|  | 71-85 | 88.5 (74.4 - 116.2) |  | 100.2 (81.4 - 129.8) |  |
| 2.5 | 61-70 | 89.9(61.8 - 116.7) | 0.39 | 105.2 (96 - 118.4) | 0.19 |
|  | 71-85 | 91.9 (76.9 - 111.9) |  | 107.4 (93.4 - 128) |  |
| 3 | 61-70 | 96.9 (66.4 - 118.9) | 0.56 | 113.3 (100.6 - 125.2) | 0.26 |
|  | 71-85 | 98.3 (85.7 - 117.9) |  | 115.2 (104.4 - 131.8) |  |
| 3.5 | 61-70 | 104.1 (73 - 123.2) | 0.42 | 121 (106.2 - 143.6) | 0.89 |
|  | 71-85 | 106.2 (95.7 - 119.8) |  | 121.4 (112.2 - 142) |  |
| Mph = miles per hour. HR = heart rate. Bpm = beats per minute. Values are presented as mean (range). P values are provided to indicate significance when bolded. | | | | | |
