# Supplementary material for: Cadence (steps/min) and relative intensity in 61 to 85-year-olds: the CADENCE-Adults study
Source: Int J Behav Nutr Phys Act. 2023 Nov 29;20:141. doi: 10.1186/s12966-023-01543-w (PMC10688086; doi:10.1186/s12966-023-01543-w)
Supplement: Supplementary file 4 — Additional file 4. Figure displaying classification accuracy of heuristic cadence thresholds and relatively-defined moderate and vigorous intensity [file 12966_2023_1543_MOESM4_ESM.docx]

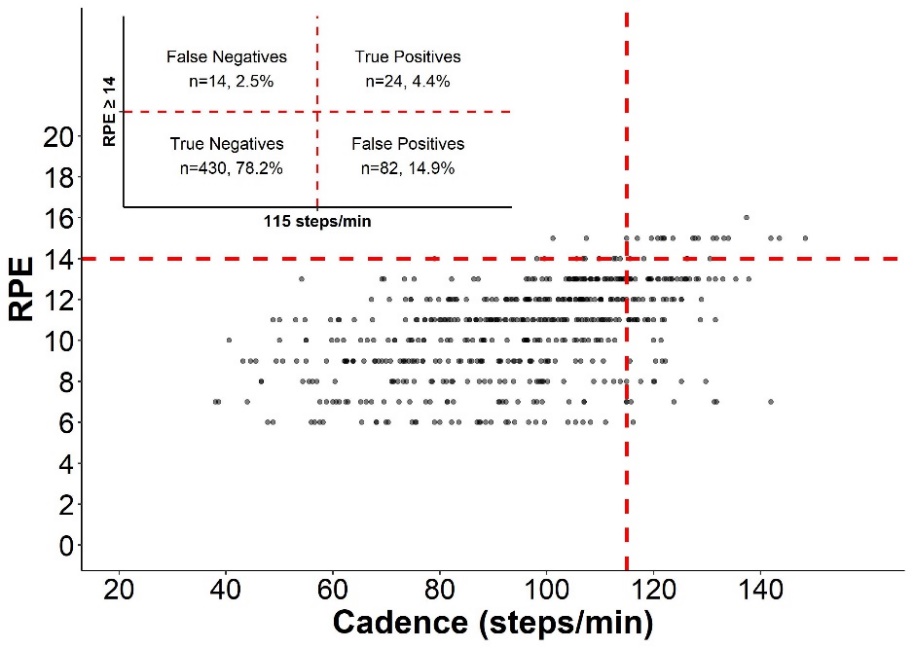

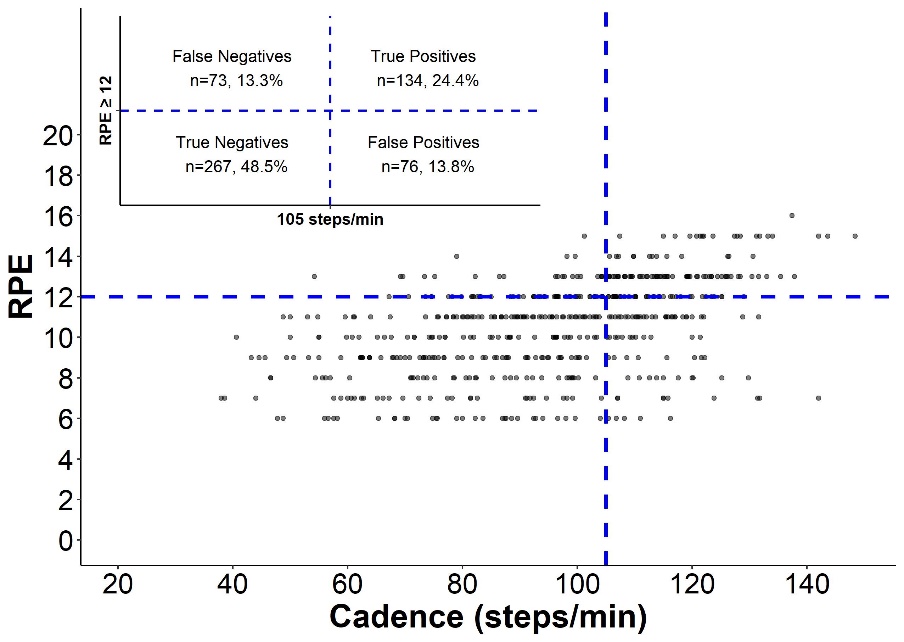

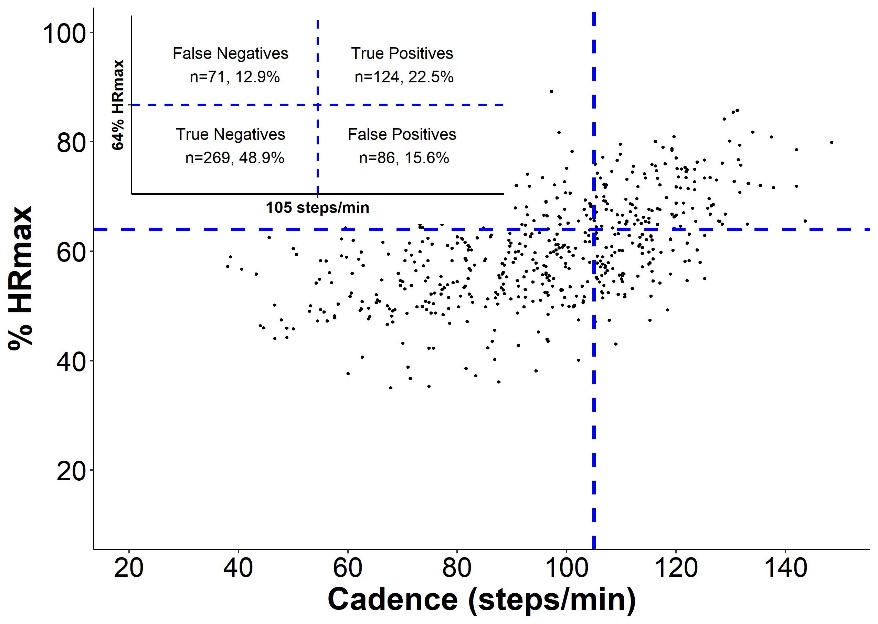

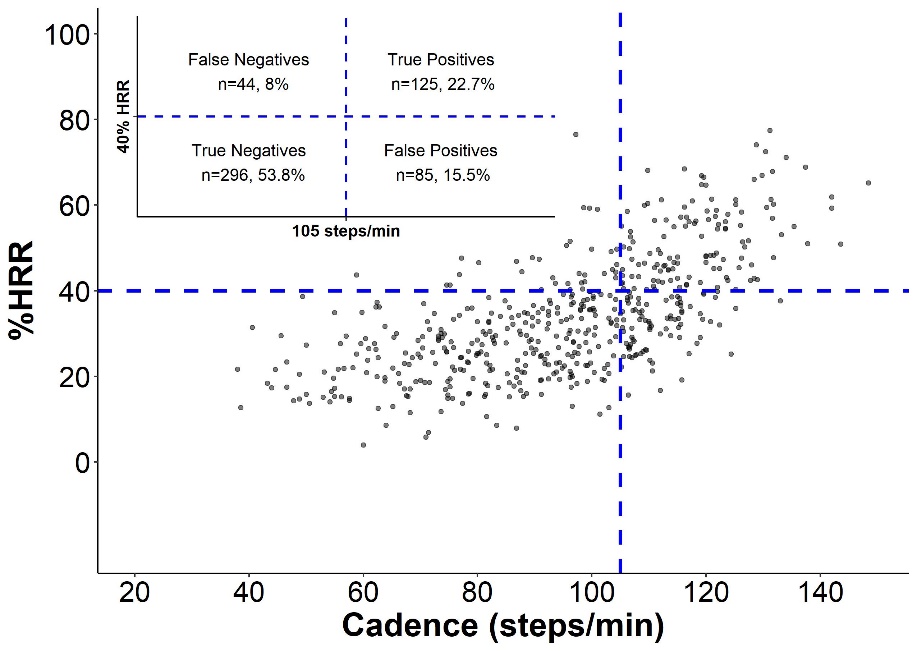

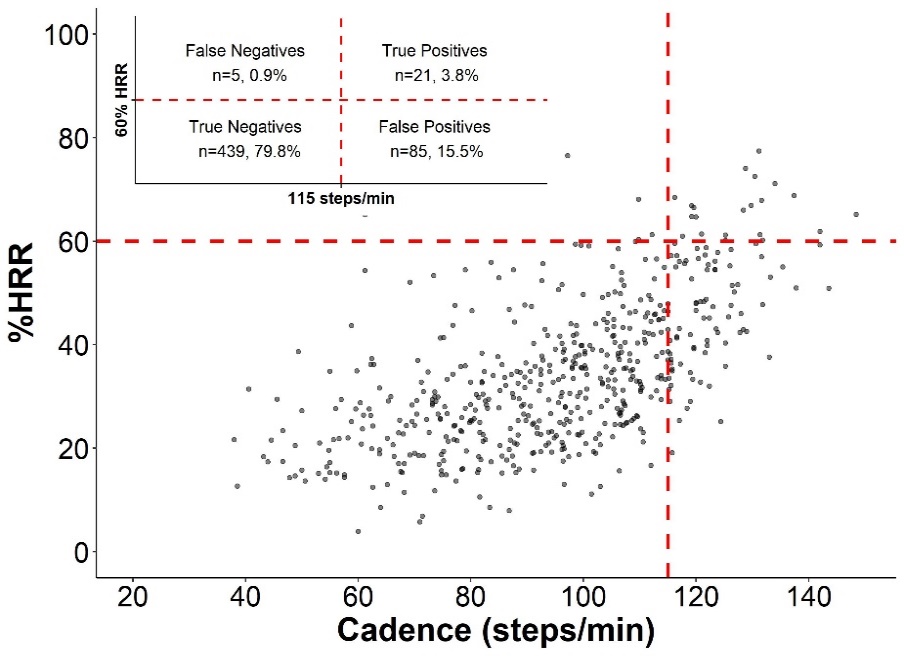

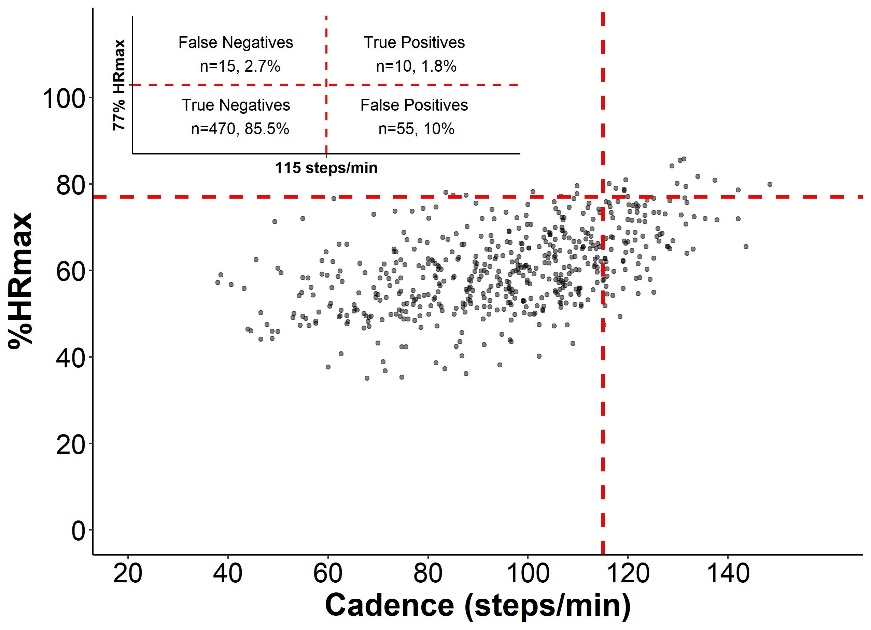


**
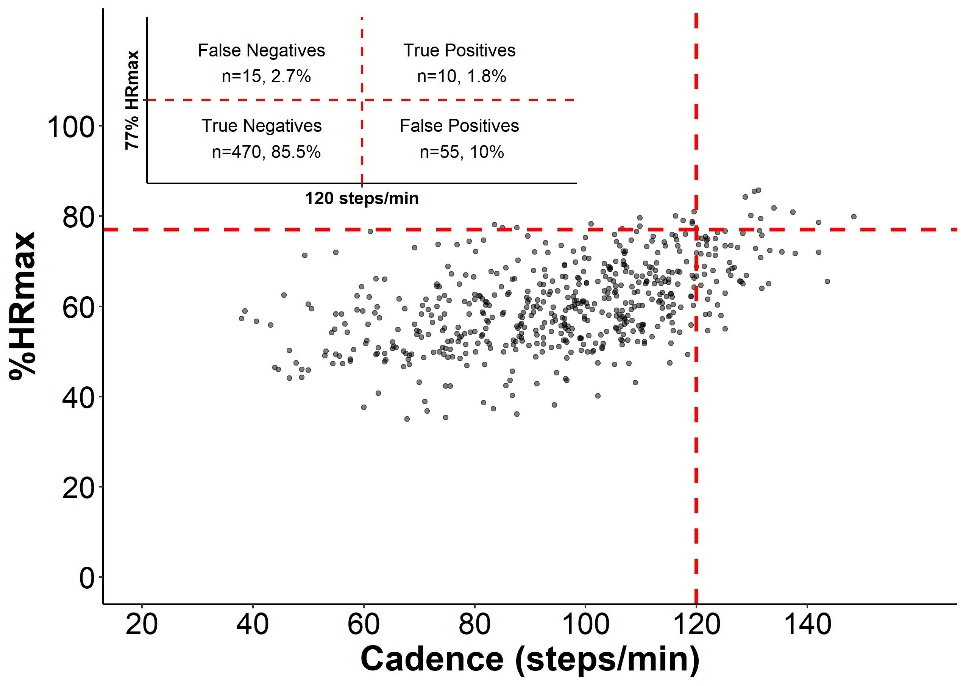
**

**
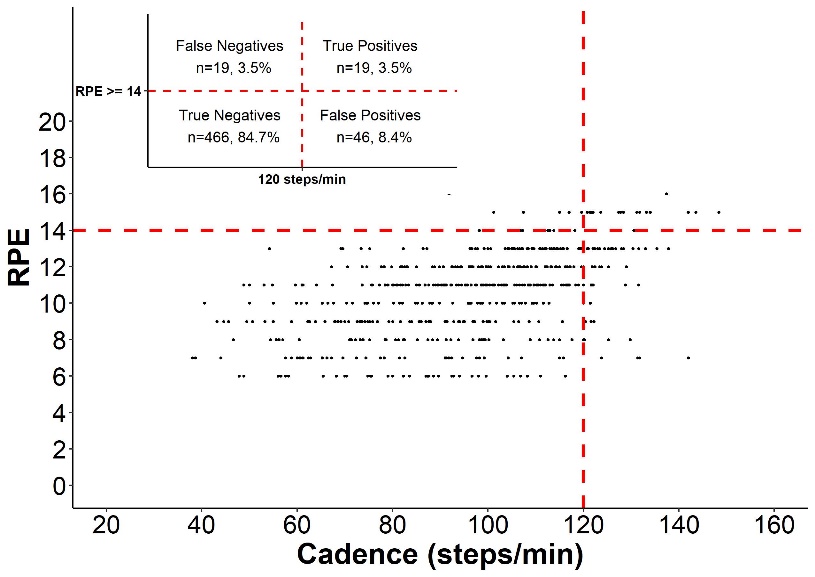

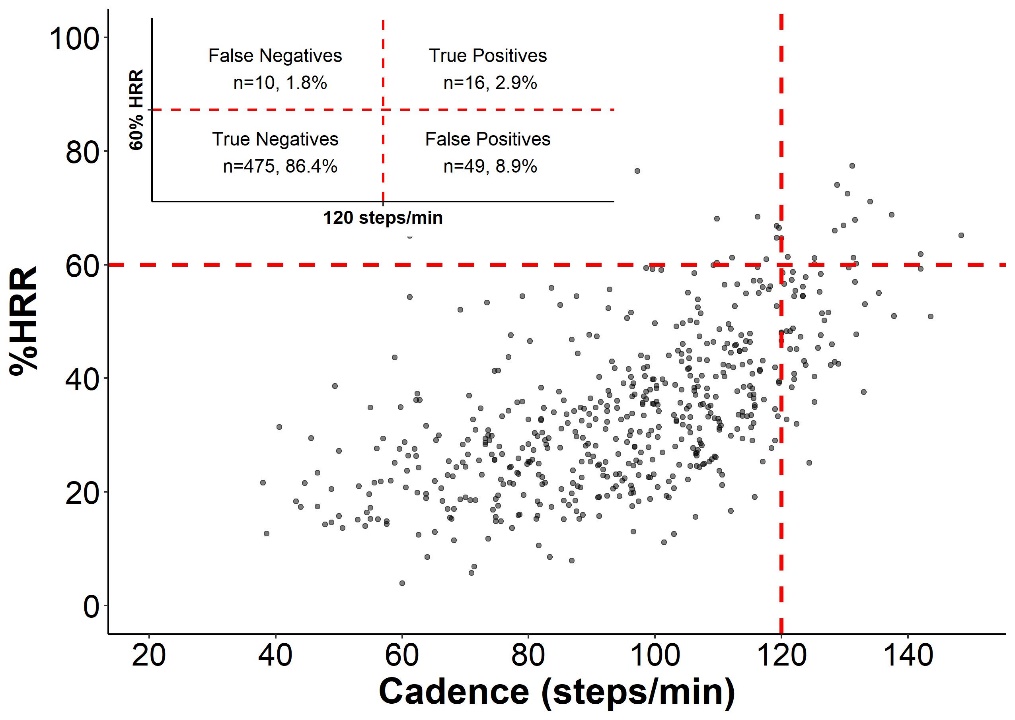
Additional file 4.** Classification accuracy of heuristic cadence thresholds and relatively-defined moderate and vigorous intensity indicators. Blue dotted lines indicate the heuristic cadence threshold (≥105 steps/min) corresponding with relatively-defined moderate intensity (≥ 64%HR maximum, ≥ 40%HR reserve, ≥ 12 RPE). Red dotted lines indicate the heuristic cadence thresholds (≥115 steps/min or ≥120 steps/min) and relatively-defined vigorous intensity (≥ 77%HR maximum, ≥ 60%HR reserve, ≥ 14 RPE). The figure displays the values for true positives, false positives, true negatives, and false negatives used to determine classification accuracy (sensitivity, specificity, positive predictive, and negative predictive values). Heart rate maximum (HRmax) = 220 - age. Heart rate reserve (HRR) = HRmax – HR resting. RPE = Rate of Perceived Exertion.
