# Supplementary material for: Cadence (steps/min) and relative intensity in 61 to 85-year-olds: the CADENCE-Adults study
Source: Int J Behav Nutr Phys Act. 2023 Nov 29;20:141. doi: 10.1186/s12966-023-01543-w (PMC10688086; doi:10.1186/s12966-023-01543-w)
Supplement: Supplementary file 5 — Additional file 5. Table with heuristic thresholds by age ranges [file 12966_2023_1543_MOESM5_ESM.docx]

**Additional file 5.** Summary of heuristic thresholds for all relatively-defined^a^ intensity indicators by age groups.

| \| **Age Range** \| **Moderate Intensity**  (≥ 64%HR_max_, ≥40%HRR, ≥12 RPE) \| **Vigorous Intensity**  (≥77%HR_max_, ≥60%HRR, ≥14 RPE) \| \| --- \| --- \| --- \| \| 21–30 years \| 120 \| 135 \| \| 31–40 years \| 120 \| 130 \| \| 41-50 years \| 115 \| 125 \| \| 51–60 years \| 110 \| 120 \| \| 61-85 years \| 105 \| 115 or 120 \|   HR maximum [HR_max_] = 220 - age. Heart rate reserve [HRR] = HR_max_ - HR_resting_. ^a^Cadence-based heuristic thresholds for relatively-defined intensity in adults 21-60 years are retrieved from McAvoy et al. (6). |
| --- | --- | --- | --- | --- | --- | --- | --- | --- | --- | --- | --- | --- | --- | --- | --- | --- | --- | --- |
